# Discharged from the emergency department following hospital-presented self-harm: referral patterns and risk of repeated self-harm

**DOI:** 10.1007/s11845-024-03722-5

**Published:** 2024-05-31

**Authors:** Grace Cully, Vincent Russell, Mary Joyce, Paul Corcoran, Caroline Daly, Eve Griffin

**Affiliations:** 1 School of Public Health, University College Cork, Cork, Ireland; 2 National Suicide Research Foundation, Cork, Ireland; 3 Department of Psychiatry, RCSI University of Medicine and Health Sciences, Beaumont Hospital, Dublin, Ireland; 4 Health Service Executive, Dublin, Ireland

**Keywords:** Assessment, Emergency department, Emergency psychiatry, Hospital services, Repetition, Self-harm

## Abstract

**Background:**

Presentation to the emergency department (ED) with self-harm provides an important opportunity for intervention.

**Aims:**

To investigate characteristics and self-harm repetition risk of those discharged from the ED without a referral for mental health–related aftercare.

**Method:**

Data on consecutive self-harm presentations to EDs for the years 2013–2019 (*n* = 55,770) were obtained from the National Self-Harm Registry Ireland. Multilevel Poisson and Cox regression models were estimated.

**Results:**

Half of the self-harm presenters were discharged from the ED (49.8%) and almost half of them did not receive a mental health–related referral (46.8%). Receipt of a psychosocial assessment was associated with a 50% reduced risk of non-referral (IRR 0.54; 95% CI 0.51–0.57). Non-referral was also less likely for young people (< 18 years), presentations involving attempted hanging, persons with previous self-harm presentations, and in the latter half of the study period (2017–2019 vs. 2013–2016), but was more likely for those brought by ambulance, presenting outside 9 am–5 pm and admitted to an ED medical assessment unit. Of those not referred, 19.3% had a repeat presentation within 12 months, compared to 22.4% of those referred. No difference in repetition risk between these two groups was evident in adjusted analyses. Self-harm history had the strongest association with repetition, with highest risk among individuals with four or more previous presentations (HR 9.30, 95% CI 8.14–10.62).

**Conclusions:**

The findings underline the importance of assessing all individuals who present with self-harm and highlight the need for comprehensively resourced 24hr services providing mental health care in the ED.

## Introduction

Hospital-presenting self-harm is one of the strongest risk factors for subsequent suicide [1]. Thus, clinical care for self-harm patients within emergency department (ED) settings is a priority, providing an important opportunity to assess the needs of the person in distress and to develop a plan for next care. Following a self-harm presentation, individuals may be admitted to a medical ward or psychiatric inpatient unit, or discharged home from the ED following medical treatment. Medical and psychiatric admissions are intensive interventions that that are not appropriate for all those who present with self-harm [2]. As such, based on data from Irish hospitals, an estimated 41–56% of self-harm patients are discharged directly from the ED [3, 4].

Clinical guidelines in Ireland and the UK recommend that individuals presenting to the hospital with self-harm are referred to appropriate follow-up services before they are discharged [5, 6]. Following ED discharge, initial aftercare may involve a mental health–related referral, including referral to psychiatric outpatient services or other mental health providers in the voluntary and community sector. Self-harm patients may also be referred for follow-up with a general practitioner. However, it is well established that care provision for individuals presenting with self-harm varies widely across hospitals [3, 4], and thus, not all patients who are discharged from the ED are likely to receive a referral for follow-up services [7, 8]. While there may be situations where a referral is considered unnecessary or inappropriate, evidence indicates that a lack of referral has the potential to be detrimental to patient safety [7, 8]. Research from the perspective of those with lived experience indicates that a lack of timely follow-up care can result in feelings of isolation, inhibited help seeking, and resistance to psychotropic treatment [8]. Furthermore, evidence from a large registry study indicates that an absence of psychiatric aftercare following hospital presented self-harm is associated subsequent mortality [9].

The decision to provide a referral to aftercare is likely to be impacted by both clinical- and service-related factors [3, 4, 7]. From a clinical perspective, individuals are likely to be prioritised for aftercare if they are perceived to be at high risk of further self-harm or suicide [7]. Referral decisions are also likely to be impacted by the presence of trained staff to complete assessments in the ED [10] and availability of aftercare appointments [7]. Patterns of referral for aftercare among those discharged home from the ED have not been thoroughly explored. Furthermore, there is an absence of research on subsequent outcomes for patients discharged without a referral. To take steps in addressing these gaps in the evidence, the objectives of this study were to examine factors associated being discharged from the ED after a self-harm presentation without a referral; and to examine risk of repeat self-harm for those discharged from the ED with and without a referral, as well as care pathways of those not discharged from the ED.

## Material and methods

### Data collection and participants

This study was conducted using data from the National Self-Harm Registry Ireland. The Registry collects data on all patients who present to all emergency departments in Ireland as a result of self-harm. Data are collected by trained data registration officers in accordance with standardised procedures. Self-harm is defined as ‘an act with non-fatal outcome in which an individual deliberately initiates a non-habitual behaviour, that without intervention from others will cause self-harm, or deliberately ingests a substance in excess of the prescribed or generally recognised therapeutic dosage, and which is aimed at realising changes that the person desires via the actual or expected physical consequences’ [11].

Analyses included patients of any age presenting during the period 1st January 2013, to 31st December 2019 (inclusive). The dataset was restricted to individuals who did not present to hospital as a result of self-harm in the 3 years before the study period (2010–2012), in order to maximise the likelihood that presentations from January, 2013, represented an individual’s first presentation. A similar approach has been used in previous studies [4, 12].

### Data items

Data items in the registry dataset include age, sex, hour and date of presentation, method(s) of self-harm, whether alcohol was consumed as part of the self-harm episode, whether the individual was brought to hospital by ambulance or other emergency services, whether the individual was admitted to a medical assessment unit in the ED, whether the individual received a psychosocial assessment by the psychiatric team in the presenting hospital, medical card status (eligibility for free healthcare based on household income), and recommended next care [13]. Information on whether a mental health nurse or non-consultant hospital doctor conducted the psychosocial assessments was not available. Method of self-harm is recorded according to the 10th revision of WHO’s International Classification of Disease codes for intentional injury (X60–X84) [14].

Recommended next care was categorised as admission to a general or psychiatric ward or unit, discharged with a mental health–related referral, or discharged without a mental health–related referral. Primary analyses focused on patients who were discharged with and without a referral. Mental health–related services that patients were recorded as being referred to included outpatient psychiatric services, community mental health teams, addiction services, and voluntary or community psychological services. Recommendations to self-refer are not considered a mental health–related referral. Presentations categorised as discharged without a referral may have received a recommendation to attend a general practitioner (GP) and may have received an emergency care plan or safety plan with details of whom to contact in crisis.

Self-harm repetition was defined as a self-harm presentation to an ED following an index self-harm presentation at any time during the 7-year study period. The study used repeat event analysis, examining all presentations (including both first and repeat presentations) as index presentations. The number of previous self-harm presentations was calculated based on the number of self-harm presentations prior to an index presentation.

Data at the level of the hospital to which a self-harm presentation was made were also used. Hospital-level data were derived from routinely available information, including the following variables: type of hospital (general or tertiary) [15], psychiatric inpatient services on the hospital site [16], liaison psychiatry team in the hospital [17], and hospital location (capital city, other city, or town).

### Data analysis

A mixed effects multilevel Poisson regression model, with robust standard errors, was fitted to identify factors associated with not being referred to mental health services among those discharged from the ED, compared to those who did receive a referral. Unadjusted and adjusted incidence rate ratios (IRR) were estimated. A Cox proportional hazard model was fitted to estimate time to self-harm repetition according to whether discharged individuals received a referral or not. Due to the fixed end date, 31 December 2019, follow-up time after an index self-harm presentation varied depending on the point during the study period that the presentation occurred, ranging from 1 to 2556 days. This variability in follow-up length was accounted for in the analyses. To account for all repeat presentations being included in the analyses, with each repeat presentation becoming an index presentation, lack of independence of observations between presentations made by the same individual was adjusted for by using robust analyses that modified the variance of estimates. For the Poisson and Cox regression models, adjusted analyses included sex and age, and individual-level variables significantly associated with the outcome variable in univariable analyses. Hospital-level variables that were significantly associated with the referral to mental health services were include in the Poisson model. Analyses were carried out using SPSS 27 and Stata 17 for Windows.

## Results

A total of 55,770 presentations to the ED for self-harm were made during the study period, involving 36,924 individuals. For half of the presentations (49.8%, *n* = 27,757), the patient was discharged from the ED following treatment. In 38.9% of presentations (*n* = 20,966, 37.6%), the individual was admitted or offered admission to a general ward or psychiatric unit in the presenting hospital, or transferred to another hospital or unit. The remaining 12.6% of presentations (*n* = 7047) involved the patient leaving the ED before receiving a recommendation or refusing admission.

Characteristics of those discharged from the ED following a self-harm presentation are presented in Table 1. Findings related to those discharged from the ED are limited to presentations where details of next care were known (96.4%, *n* = 26,765). More than half of those discharged from the ED were female (55.6%), the median age was 29 years (interquartile range 22), and 43.8% had a medical card (Table 1). The most common method of self-harm was intentional drug overdose (IDO, 53.3%), followed by self-cutting (23.3%), and alcohol was involved in 32.0% of presentations. The majority of the individuals discharged from the ED received a psychosocial assessment (84.2%), half (49.4%) were brought in by ambulance or other emergency services, and 31.2% were admitted to a medical assessment unit in the ED.

**Table 1:** Characteristics of self-harm presentations that were discharged from the ED without a referral

	**All discharged** **(**26,765, 49.5%)	
	***n***	**%**
**Sex**		
Male	11,884	44.4
Female	14,881	55.6
**Age**		
< 18 years	5382	20.1
19–24 years	6446	24.1
25–44 years	10,254	38.3
45–64 years	4135	15.5
> 65 years	548	2.1
**Method**		
Intentional drug overdose (IDO) only	14,264	53.3
Self-cutting only	6240	23.3
IDO and self-cutting	1343	5.0
Attempted hanging only	1366	5.1
Attempted drowning only	679	2.5
Other	2873	10.7
**Alcohol involved**	8559	32.0
**Brought in by ambulance**	13,007	49.4
**Admission to MAU**^**a**^	7579	31.2
**Received psychosocial assessment**^**b**^	20,344	84.2
**Presented at weekend**	7693	28.7
**Presented outside of 9 am–5 pm**	17,181	64.4
**Previous self-harm presentations**	7933	29.6
**Medical card status**	8869	43.8
**Year of presentation**		
2013–2016	13,829	51.7
2017–2019	12,936	48.3

^a^Admission to MAU unknown for 6.6% of presentations

^b^Assessment unknown for 7.5% of presentations

Of those discharged from the ED, 53.3% (*n* = 14,252) received a referral. The most common type of referral was to outpatient psychiatric services (70.4%, *n* = 10,035), followed by community mental health team (17.9%; *n* = 2,550), psychological services (7.1%, *n* = 1,014), and addiction services (4.6%, *n* = 653). Of the 12,513 (46.8%) presentations that did result in a referral to mental health services, 39.8% were referred to a general practitioner (*n* = 4985).

### Factors associated with ED discharge without a referral

The characteristics of those discharged from the ED, with and without a referral, are presented in Table 2. Table 2 also details the results from crude and adjusted multilevel Poisson regression models comparing individuals discharged from the ED without a referral with those discharged with a referral. Psychosocial assessment was the factor most strongly associated with being referred to mental health services, with those assessed having half the risk of being discharged without a referral compared to those who were not assessed (incidence rate ratio (IRR) 0.54; 95% confidence interval (CI) 0.51–0.57). Adults were more likely to be discharged from the ED without a referral, compared to those aged under 18 years (1.40 1.30–1.51). Attempted hanging as the method of self-harm was associated with reduced risk of discharge without a referral compared to all other self-harm methods (0.80; 0.70–0.90). Persons with a history of previous self-harm presentations were also less likely to be discharged without a referral (0.93; 0.88–0.98).

**Table 2:** Multilevel Poisson regression models with individual-level and hospital-level factors associated with not receiving a mental health–related referral prior to discharge from the ED following self-harm

	**Discharged without referral** (12,513, 46.8%)		**Discharged with referral** (14,252, 53.3%)		**Factors associated with being** **discharged without a referral**			
	***n***	**%**	***n***	**%**	**Unadjusted IRR (95% CI)**	***p*-value**	**Adjusted IRR (95% CI)**	***p*-value**
***Individual-level factors ***^***a***^								
**Male** (ref, female)	5904	47.2	6609	52.8	1.12 (1.08–1.16)	< 0.001	1.04 (1.00–1.08)	0.060
**Age > 18 years** (ref, < 18 years)	11,904	83.5	10,905	87.2	1.18 (1.12–1.24)	< 0.001	1.33 (1.25–1.42)	< 0.001
**Method**								
Intentional drug overdose (IDO) only (ref)	6963	55.7	7301	51.2	1		1	
Self-cutting only	2993	23.9	3247	22.8	0.98 (0.94–1.03)	0.421	0.96 (0.91–1.01)	0.123
IDO and self-cutting	508	4.1	835	5.9	0.77 (0.71–0.85)	< 0.001	0.88 (0.80–0.97)	0.013
Attempted hanging only	504	4.0	862	6.1	0.76 (0.69–0.83)	< 0.001	0.78 (0.70–0.87)	< 0.001
Attempted drowning only	299	2.4	380	2.7	0.90 (0.80–1.01)	0.081	0.93 (0.81–1.07)	0.323
Other	1246	10.0	1627	11.4	0.89 (0.84–0.94)	< 0.001	0.94 (0.88–1.01)	0.086
**Alcohol involved** (ref, no)	3820	30.5	4739	33.3	0.93 (0.90–0.97)	0.001	1.04 (0.99–1.09)	0.083
**Brought in by ambulance** (ref, no)	5905	48.2	6976	49.6	0.95 (0.92–0.99)	0.008	1.08 (1.03–1.13)	< 0.001
**Admission to medical assessment unit** (ref, no)^***b***^	3667	35.9	10,139	72.2	1.23 (0.18–1.28)	< 0.001	1.17 (1.10–1.24)	< 0.001
**Received psychosocial assessment** (ref, no)^***c***^	7428	73.8	12,916	91.5	0.53 (0.51–0.55)	< 0.001	0.51 (0.49–0.57)	< 0.001
**Presented at weekend** (ref, no)	3758	30.0	3935	27.6	1.06 (1.02–1.11)	0.001	1.03 (0.99–1.08)	0.153
**Presented outside of 9 am–5 pm** (ref, presented from 9 am–5 pm)	8375	67.1	5395	38.0	1.13 (1.09–1.17)	< 0.001	1.08 (1.04–1.13)	< 0.001
**Previous self-harm presentations** (ref, no)	3439	27.5	4494	31.5	0.90 (0.87–0.94)	< 0.001	0.89 (0.88–0.98)	< 0.001
**Year of presentation**								
2013–2016 (ref)	6837	54.6	6992	49.1	1		1	
2017–2019	5676	45.4	7260	50.9	0.89 (0.86–0.92)	< 0.001	0.86 (0.85–0.93)	< 0.001
***Hospital-level factors ***^***d***^								
**Hospital admission rate for self-harm** (ref, low < 0.19)								
Medium (0.19–0.26)					0.99 (0.95–1.03)	0.468	1.26 (0.88–1.81)	0.114
High (> 0.26)					1.35 (0.30–1.42)	< 0.001	1.35 (0.93–1.95)	0.114
**Psychiatric in-patient facilities** (ref, offsite)					1.12 (1.07–1.18)	< 0.001	0.98 (0.68–1.41)	0.924
**Hospital location** (ref, other city)								
Dublin City					0.88 (0.85–0.92)	< 0.001	1.00 (0.63–1.57)	0.986
Town					0.75 (0.72–0.78)	< 0.001	0.57 (0.28–1.15)	0.115
**Liaison psychiatry services** (ref, no)					1.31 (1.26–1.37)	< 0.001	0.66 (0.62–1.47)	0.114
**Model 4 hospital** (ref, model 3)					0.90 (0.86–0.93)	< 0.001	0.96 (0.34–1.63)	0.836

*IRR* incidence rate ratio. All variables listed in the table are included in the adjusted model

^a^Variables are at the level of the individual presentation

^b^Admission to MAU unknown for 6.63% of presentations

^c^Assessment unknown for 7.5% of presentations

^d^Variables are at the level of the hospital to which a self-harm presentation was made

Those admitted to a medical assessment unit in the ED had higher risk of ED discharge without a referral (1.10; 1.03–1.18) compared to persons not admitted to an MAU. Arriving by ambulance (1.09; 1.04–1.14) and presenting to the ED outside of the hours 9 am to 5 pm (1.08 (0.92–1.01)) also conferred increased risk of discharge without a referral. Presentations resulting in admission to a MAU and arriving by ambulance were significantly more likely to involve adults, alcohol consumption, intentional drug overdose, and not self-cutting (Supplementary Table 1). Those arriving by ambulance were also more likely to present out of hours, to have a previous history of self-harm, and to have a medical card. Male sex was associated with ambulance presentation, while female sex was associated with admission to MAU.

While there was evidence of an association of hospital-level factors with referral patterns post-discharge in crude analyses, these associations did not remain in the adjusted model.

### Risk of repeat self-harm

Of those discharged from the ED, 20.9% of episodes were followed by a repeat self-harm presentation within 12 months. Of those discharged without a mental health referral, 19.3% had a repeat presentation, compared to 22.4% of those who received such a referral. Crude analyses indicated that repetition risk was lower for those who did not receive a referral compared to those who were referred to mental health services (hazard ratio (HR) 0.83; 95% CI 0.78–0.90). However, there was no statistical evidence for a difference in repetition risk between these two groups in adjusted analyses (0.95; 0.88–1.02) (Table 3). In the adjusted model, self-harm history had the strongest association with subsequent repetition (Table 3). Repetition risk increased with each additional previous self-harm presentation, with the highest risk following presentations among those with a history of four or more previous presentations (adjusted hazard ratio (AHR) 9.30, 95% CI 8.14–10.62). Method of self-harm was also associated with repetition risk, with presentations involving self-cutting associated with higher risk of repetition compared to presentations IDO only.

**Table 3:** Adjusted Cox regression model for repeat self-harm within 12 months

	**Repeat self-harm** (*n* = 19,224) ^a^	
	HR (95% CI)	*p*-value
**Care pathways**		
ED discharge without referral	0.95 (0.88–1.02)	0.134
ED discharge with referral	Ref	
**Male** (ref, female)	0.92 (0.84–1.00)	0.055
**Age > 18 years** (ref, < 1 8 years)	0.91 (0.83–1.00)	0.052
**Method**		
Self-cutting only	1.45 (1.32–1.60)	< 0.001
IDO and self-cutting	1.41 (1.23–1.61)	< 0.001
Attempted hanging only	0.91 (0.77–1.08)	0.271
Attempted drowning only	0.85 (0.66–1.09)	0.196
Other	1.23 (1.10–1.37)	< 0.001
**Received psychosocial assessment** (ref, no)	0.95 (0.87–1.04)	0.238
**Presented outside of 9 am–5 pm** (ref, presented from 9am-5pm)	1.01 (0.94–1.08)	0.804
**Number previous self-harm presentations** (ref, none)		
One	2.34 (2.15–2.55)	< 0.001
Two	3.85 (3.47–4.28)	< 0.001
Three	4.72 (4.16–5.35)	< 0.001
Four or more	9.30 (8.14–10.62)	< 0.001
**Medical card holder** (ref, not medical card holder)	1.23 (1.15–1.33)	< 0.001
**Year of presentation** (ref, 2013–2016)	0.98 (0.91–1.05)	0.512

^a^Analysis based on presentations that had complete data for all variables included in the model

## Discussion

Our findings indicate that one in two individuals who presented to the ED with self-harm was discharged following medical treatment. Almost half of those discharged from the ED were not referred for follow-up with mental health services. The findings indicate that not receiving a psychosocial assessment was the factor that most strongly predicted not receiving a referral. Other factors that increased the likelihood of discharge without referral were admission to a medical assessment unit in the ED, arriving by ambulance, and presenting outside of the hours 9 am to 5 pm. Being under 18 years of age, attempted hanging as method of self-harm, history of previous self-harm presentations, and presenting in the latter half of the study period were associated with decreased risk of non-referral.

No previous studies have directly focused on the profile of those discharged from the ED without a referral. A study on five hospitals in England reported on referral post discharge from the ED or a medical ward and found that 31% of patients were referred to outpatient mental health services [18], which was lower than the 53% referral rate in the present study. Given that admission for treatment in a medical assessment unit in the ED in the present study was associated with not being referred, it is possible that the lower proportion reported by Steeg et al. (2018) is related to the inclusion of patients who were admitted medically. A stay in hospital following an ED presentation, whether in an ED assessment unit or a medical ward, may lead to a lower likelihood of referral due to a de-escalation of the crisis during that period [2], or could be related to clinical responsibility of the patient being passed to the medical teams. Consistent with our study, Steeg and colleagues reported that a history of self-harm was associated with receiving a referral upon discharge.

The strong association between referral and assessment in our study shows that where patients receive an assessment, they are likely to be referred to next care. This indicates that there is a good understanding of the importance of referral among those providing care in ED, but that one of the problems may be ensuring that all patients are assessed. Patients are more likely to be discharged without an assessment when they present out of hours [19, 20], which we also identified as a predictor of being discharged without a mental health referral. Furthermore, those presenting via ambulance, most often occurring out of hours, were less likely to be referred to mental health–related follow-up care. These factors indicate the need for comprehensively resourced 24hr services for the mental health care of self-harm patients in the ED. The model of care of the National Clinical Programme for Self-Harm and Suicide-related Ideation (NCPSHI) in Ireland [21], which underpins the care of those presenting to the ED with self-harm, specifies that the components of the model should be delivered 24hr a day, 7 days a week. However, in some acute hospitals in the country, there is no onsite psychiatry cover out of normal working hours. Where out-of-hour cover is provided, it is the responsibility of on-call psychiatry trainees on a short-term rotation, with access to supervision from an on-call consultant [22]. Thus, the importance of providing regular training in the assessment and care of self-harm patients to non-consultant hospital doctors is of the utmost importance to achieve high quality care out of hours.

The level of referral to mental health care may also be related to the accessibility and availability of services to refer to. Indeed, the implementation plan of Ireland’s mental health strategy, Sharing the Vision [23], highlights the need to improve access routes into mental health services to ensure comprehensive and integrated services for the provision of mental healthcare are available in acute hospitals. A recent qualitative study in the UK examined factors underlying the decision to not refer persons attending the ED in crisis to mental health services [24]. The pressure on under resourced mental health services was highlighted, suggesting that clinicians in the ED have to take on a role as gatekeeper of mental health services, rationing referrals to those that are deemed most in need [24]. Indeed, the decreased risk of non-referral among those presenting with higher lethality methods of self-harm, with repeat self-harm and those aged under 18, indicates that referrals may be prioritised for those who are perceived to be at higher risk for further self-harm or suicide. However, guidelines in Ireland and the UK recommend that prediction of risk should not be relied on to allocate treatment [2, 21, 25, 26]. Aftercare planning should be based on a comprehensive assessment of the individual needs and should be collaborative with the patient and their family. In this study, one in four of those who presented to the ED with self-harm was discharged without a referral and one in five of those individuals re-presented within 12 months. This indicates continued distress and a gap in the continuity of care for these individuals. Repeated acts of self-harm are associated with increased risk of subsequent suicide [18], highlighting that there may be missed opportunities for intervention among those discharged from the ED without referral.

An increase in referrals was observed over the study period and may be related to implementation of the aforementioned national clinical programme for the care of self-harm in the ED across the majority of hospitals in the country, beginning in 2015 [21]. Indeed, a recent evaluation found that the introduction of the clinical programme was associated with a significant increase in referral, particularly in hospitals that had limited pre-existing resources for caring for those presenting with self-harm. Consistent with this, the availability of dedicated staff has been shown to be associated with improved care for self-harm in an ED in the UK [27]. Central components of the clinical programme include the provision of a biopsychosocial assessment and appropriate follow-up and referral to secondary mental healthcare, as well aspects it was not possible to capture in this study including the provision of an emergency care plan, collaboration with next of kin, and the provision of a 24hr phone call. As such, individuals discharged from the ED without a referral may have received thorough follow-up from the hospital that we have not captured with the data available for this study. It is clear that general practitioners also play an important role in mental healthcare and support for this group of self-harm patients. Those discharged without a referral to mental health services were frequently referred to a general practitioner as their only point of aftercare. Studies examining GPs’ experiences of managing suicidal patients have identified barriers including a potential lack of confidence in the management of suicidal patients, structural inadequacies in mental health service provision, and difficulties in assessing suicide risk [28, 29]. The NCPSHI has extended its reach beyond the ED to increase capacity in primary care with the aim of decreasing the number of patients presenting to the ED whose needs could be met in the community. This involves the development of a Suicide Crisis Assessment Nurse (SCAN) service located within primary care [21] to provide assessments to patients in suicidal crisis without a medical need. In light of the findings of the current study, increased capacity to assess individuals in crisis has the potential to have an impact on the rates of ED discharge without referral.

Research on the links between ED services for self-harm patients and community mental health teams, addiction services, and other psychological services is limited. Indeed, the information on next care in this study reflects the decision of a clinician to refer or make a recommendation. It does not reflect attendance at appointments or receipt of treatment as that information is not available. As such, when examining patient outcomes, it is important to note that those discharged without a referral may seek out further support with mental health services themselves and those who are referred may not engage in the recommended follow-up. Thus, tracking attendance at follow-up appointments for self-harm patients is an important future avenue of research, to make investigations of patient outcomes more robust, and to inform clinicians which patients are likely to not engage and may require bridging to next care.

Compared to those discharged with a referral, individuals who were not referred were less likely to make a repeat presentation within 12 months. The pattern of higher repetition following referral may be due to confounding by indication, whereby those most likely to re-present are also those more likely to be allocated care [18, 30, 31]. In the current study, in adjusted analyses, there is no difference in repetition risk between those discharged with and without a referral. The factor that was most strongly related to self-harm repetition was a person’s previous history of self-harm presentations, with repetition risk increasing with each additional previous self-harm presentation, consistent with existing evidence [12]. Considering that those with a history of self-harm were also more likely to receive a referral, these findings provide further evidence that mental health care was allocated to individuals with highest risks of repeated self-harm. This is supported by previous studies of hospital presented self-harm that have used propensity score methods to adjust for differences between those who did and did not receive a referral, and found associations with adverse outcomes diminished in propensity adjusted analyses [18, 30, 31].

A strength of this study is the data used, from a national self-harm registry, including all acute hospitals in the country. Using multilevel modelling techniques to account for random variation across hospitals, as well as fixed hospital-related hospital factors and individual factors, is a further strength. However, potentially important clinical data were not available, including information on psychiatric diagnoses and history of engagement with mental health services, information on assessment and next care for those transferred to another hospital following their ED presentation, and data on repetition of self-harm that did not result in a presentation to the ED.

Those who present to the hospital with self-harm are likely to require further support after they are discharged from the ED [2, 8]. As such, guidelines for the clinical management of self-harm in the ED recommend that all self-harm patients receive a plan for appropriate aftercare, which may include outpatient mental health services or services within the voluntary or community sectors [2, 21]. The findings of this study indicate that not all those who present to the ED with self-harm receive such a plan. The findings highlight potential areas for enhancement of the care provided to self-harm patients, particularly in out-of-hours service provision. The observed increase in referral for those discharged from the ED over the study period suggests an improvement in implementation of best practice guidelines over time.

## Data Availability

Access to data from the National Self-Harm Registry Ireland may be requested by contacting the National Suicide Research Foundation ( info@nsrf.ie ). Access to the syntax used for this study can be requested from the corresponding author ( grace.cully@ucc.ie ).

## References

[1] Carroll R, Metcalfe C, Gunnell D (2014) Hospital presenting self-harm and risk of fatal and non-fatal repetition: systematic review and meta-analysis. PLoS ONE 9(2):e89944 24587141 10.1371/journal.pone.0089944 PMC3938547

[2] NICE (2022) Self-harm: assessment, management and preventing recurrence [NICE guideline NG225]. London: National Institute for Health and Care Excellence. https://www.nice.org.uk/guidance/ng225/resources/selfharm-assessment-management-and-preventing-recurrence-pdf-66143837346757. Accessed 29 May 2024 36595613

[3] Arensman E, Griffin E, Daly C et al (2018) Recommended next care following hospital-treated self-harm: patterns and trends over time. PLoS ONE 13(3):e0193587 29494659 10.1371/journal.pone.0193587 PMC5832269

[4] Griffin E, Gunnell D, Corcoran P (2020) Factors explaining variation in recommended care pathways following hospital-presenting self-harm: a multilevel national registry study. BJPsych Open 6(6). 10.1192/bjo.2020.116 10.1192/bjo.2020.116 PMC7745229 33234189

[5] National Collaborating Centre for Mental Health (UK) (2004) Self-Harm: The Short-Term Physical and Psychological Management and Secondary Prevention of Self-Harm in Primary and Secondary Care. Leicester (UK): British Psychological Society (UK). PMID: 21834185 21834185

[6] Wrigley M, Jennings R, MacHale S, Cassidy E (2017) Assessment and management of self harm in emergency departments in Ireland: the national clinical programme. Int J Integr Care 17(5):312. 10.5334/ijic.3629

[7] Quinlivan L, Gorman L, Marks S et al (2023) Liaison psychiatry practitioners’ views on accessing aftercare and psychological therapies for patients who present to hospital following self-harm: multi-site interview study. BJPsych Open 9(2):e34 36803955 10.1192/bjo.2023.2 PMC9970172

[8] Cully G, Leahy D, Shiely F, Arensman E (2020) Patients’ experiences of engagement with healthcare services following a high-risk self-harm presentation to a hospital emergency department: a mixed methods study. Arch Suicide Res 26(1):91-111.1–21. 10.1080/13811118.2020.1779153 32576083 10.1080/13811118.2020.1779153

[9] Qin P, Hawton K, Mortensen PB, Webb R (2014) Combined effects of physical illness and comorbid psychiatric disorder on risk of suicide in a national population study. Br J Psychiatry 204(6):430–435 24578445 10.1192/bjp.bp.113.128785

[10] Cully G, Corcoran P, Gunnell D and others (2023) Evaluation of a national clinical programme for the management of self-harm in hospital emergency departments: impact on patient outcomes and the provision of care. BMC Psychiatry 23(1):917 38062378 10.1186/s12888-023-05340-4 PMC10701986

[11] Schmidtke A, Bille-Brahe U, DeLeo D, Kerkhof A, Bjerke T, Crepet P et al (1996) Attempted suicide in Europe: rates, trends and sociodemographic characteristics of suicide attempters during the period 1989–1992. Results of the WHO/EURO Multicentre Study on Parasuicide. Acta Psychiatr Scand. 93(5):327–338 8792901 10.1111/j.1600-0447.1996.tb10656.x

[12] Cully G, Corcoran P, Leahy D and others (2019) Method of self-harm and risk of self-harm repetition: findings from a national self-harm registry. J Affect Disord 246:843–850 30795489 10.1016/j.jad.2018.10.372

[13] Joyce M, Chakraborty S, O’Sullivan P, Daly C, McTernan N, Nicholson S et al (2022) National Self-Harm Registry Ireland annual report 2020. Cork: National Suicide Research Foundation. https://www.nsrf.ie/wp-content/uploads/2022/11/NSRF-National-Self-Harm-Registry-Ireland-annual-report-2020-Final-for-website.pdf. Accessed 29 May 2024

[14] Organization WH (2004) ICD-10: international statistical classification of diseases and related health problems : tenth revision [Internet]. World Health Organization; [cited 2021 Apr 29]. Available from: https://apps.who.int/iris/handle/10665/42980

[15] Health Service Executive (2014) Securing the future of smaller hospitals: a framework for development [Internet]. Dublin: Health Service Executive. Available from: https://assets.gov.ie/12170/91124d282ee84248b929698e050dedc5.pdf

[16] Health Service Executive (2018) Management Data Report December 2018 [Internet]. Dublin: Health Service Executive. Available from: https://www.hse.ie/eng/services/publications/performancereports/management-data-report-december-2018.pdf

[17] Health Service Executive (2017) National clinical programme for the assessment and management of patients presenting to the emergency department following self-harm: review of the operation of the programme 2017. Dublin: Health Service Executive, Mental Health Division. https://www.hse.ie/eng/services/publications/clinical-strategy-and-programmes/national-clinical-programme-for-the-assessment-and-management-of-patients-presenting-to-emergency-departments-following-self-harm.pdf. Accessed 29 May 2024

[18] Steeg S, Carr M, Emsley R, Hawton K, Waters K, Bickley H et al (2018) Suicide and all-cause mortality following routine hospital management of self-harm: propensity score analysis using multicentre cohort data. PLOS ONE 13(9):e0204670. 10.1371/journal.pone.0204670 30261030 10.1371/journal.pone.0204670 PMC6161837

[19] Bennewith O, Peters TJ, Hawton K et al (2005) Factors associated with the non-assessment of self-harm patients attending an accident and emergency department: results of a national study. J Affect Disord 89(1–3):91–97 16226810 10.1016/j.jad.2005.08.011

[20] Kapur N, Murphy E, Cooper J and others (2008) Psychosocial assessment following self-harm: results from the multi-centre monitoring of self-harm project. J Affect Disord 106(3):285–293 17761308 10.1016/j.jad.2007.07.010

[21] National Clinical Programme for Self Harm and Suicide Related Ideation – Implementation Advisory Group (2022) National clinical programme for self-harm and suicide-related ideation: updating the national clinical programme for the assessment and management of patients presenting to the emergency department following self-harm. Dublin: Health Service Executive. https://www.hse.ie/eng/about/who/cspd/ncps/self-harm-suicide-related-ideation/moc/mhncp-self-harm-model-of-care.pdf. Accessed 29 May 2024

[22] Doherty AM, Plunkett R, McEvoy K and others (2021) Consultation-liaison psychiatry services in Ireland: a national cross-sectional study. Front Psychiatry 29(12):748224 10.3389/fpsyt.2021.748224 PMC8666631 34912252

[23] Department of Health, Health Service Executive (2022) Implementation plan 2022–2024: sharing the vision [Internet]. Dublin: The Statutory Office; [cited 2024 May 17]. Available from: https://www.gov.ie/en/publication/2e46f-sharing-the-vision-a-mental-health-policy-for-everyone/

[24] Bergen C, Lomas M, Ryan M, McCabe R (2023) Gatekeeping and factors underlying decisions not to refer to mental health services after self-harm: triangulating video-recordings of consultations, interviews, medical records and discharge letters. SSM - Qual Res Health 27:100249

[25] Graney J, Hunt IM, Quinlivan L and others (2020) Suicide risk assessment in UK mental health services: a national mixed-methods study. Lancet Psychiatry 7(12):1046–1053 33189221 10.1016/S2215-0366(20)30381-3

[26] Lucey JV, Matti B (2023) Suicide risk assessment: time to think again? Irish J Psychol Med 40(3):323–325 10.1017/ipm.2021.76 34847969

[27] Jackson J, Nugawela MD, De Vocht F and others (2020) Long-term impact of the expansion of a hospital liaison psychiatry service on patient care and costs following emergency department attendances for self-harm. BJPsych Open 6(3):e34 32238204 10.1192/bjo.2020.18 PMC7176831

[28] Leavey G, Mallon S, Rondon-Sulbaran J et al (2017) The failure of suicide prevention in primary care: family and GP perspectives–a qualitative study. BMC Psychiatry 17(1):369 29157221 10.1186/s12888-017-1508-7 PMC5697339

[29] Saini P, While D, Chantler K, Windfuhr K, Kapur N (2014) Assessment and management of suicide risk in primary care. Crisis. 35(6):415–25. 10.1027/0227-5910/a000277 25234744 10.1027/0227-5910/a000277

[30] Steeg S, Emsley R, Carr M et al (2018) Routine hospital management of self-harm and risk of further self-harm: propensity score analysis using record-based cohort data. Psychol Med 48(2):315–326 28637535 10.1017/S0033291717001702

[31] Cully G, Corcoran P, Leahy D and others (2021) Factors associated with psychiatric admission and subsequent self-harm repetition: a cohort study of high-risk hospital-presenting self-harm. J Ment Health 30(6):751–759 34749587 10.1080/09638237.2021.1979488

